# A genetic intervention

**DOI:** 10.7554/eLife.72000

**Published:** 2021-08-12

**Authors:** Colin Sutherland, Didier Menard

**Affiliations:** 1 Department of Infection Biology, Faculty of Infectious and Tropical Diseases, London School of Hygiene and Tropical Medicine London United Kingdom; 2 Malaria Genetics and Resistance Unit, INSERM U1201 Paris France; 3 Department of Parasites and Insect Vectors, Institut Pasteur Paris France

**Keywords:** malaria, genetic surveillance, drug resistance, Asia, *P. falciparum*

## Abstract

A tool that analyzes the genome of parasites found in the blood of malaria patients can help inform policy decisions on how best to tackle the rise in drug-resistant infections.

**Related research article** Jacob CG, Thuy-Nhien N, Mayxay M, Maude RJ, Quang HH, Hongvanthong B, Vanisaveth V, Ngo Duc T, Rekol H, van der Pluijm R, von Seidlein L, Fairhurst R, Nosten F, Hossain MA, Park N, Goodwin S, Ringwald P, Chindavongsa K, Newton P, Ashley E, Phalivong S, Maude R, Leang R, Huch C, Dong LT, Nguyen KT, Nhat TM, Hien TT, Nguyen H, Zdrojewski N, Canavati S, Sayeed AA, Uddin D, Buckee C, Fanello CI, Onyamboko M, Peto T, Tripura R, Amaratunga C, Myint Thu A, Delmas G, Landier J, Parker DM, Chau NH, Lek D, Suon S, Callery J, Jittamala P, Hanboonkunupakarn B, Pukrittayakamee S, Phyo AP, Smithuis F, Lin K, Thant M, Hlaing TM, Satpathi P, Satpathi S, Behera PK, Tripura A, Baidya S, Valecha N, Anvikar AR, Ul Islam A, Faiz A, Kunasol C, Drury E, Kekre M, Ali M, Love K, Rajatileka S, Jeffreys AE, Rowlands K, Hubbart CS, Dhorda M, Vongpromek R, Kotanan N, Wongnak P, Almagro Garcia J, Pearson RD, Ariani CV, Chookajorn T, Malangone C, Nguyen T, Stalker J, Jeffery B, Keatley J, Johnson KJ, Muddyman D, Chan XHS, Sillitoe J, Amato R, Simpson V, Gonçalves S, Rockett K, Day NP, Dondorp AM, Kwiatkowski DP, Miotto O. 2021. Genetic surveillance in the Greater Mekong subregion and South Asia to support malaria control and elimination. *eLife* **10**:e62997. doi: 10.7554/eLife.62997

Malaria cases may have decreased over the last 15 years, but the number of parasites resistant to treatment is rising, particularly in Southeast Asia. Indeed, some *Plasmodium falciparum* parasites (which cause the most severe form of the disease) no longer respond to the most widely used antimalarial drugs (Blasco et al., 2017; Menard and Dondorp, 2017). This includes artemisinin-based combination therapy (ACT), the first line of defense recommended by National Malaria Control Programs (Noedl et al., 2008; Dondorp et al., 2009; Cheeseman et al., 2012; Ariey et al., 2014; Takala-Harrison et al., 2015; Amato et al., 2018; Hamilton et al., 2019; Imwong et al., 2020; Stokes et al., 2021). The reduced susceptibility to artemisinin and its partner drugs has resulted in ACT therapy failing to treat over 50% of malaria cases in some regions of Cambodia, Thailand and Vietnam (van der Pluijm et al., 2020; van der Pluijm et al., 2019).

In order to make rapid, effective decisions on how best to tackle this rise in resistance, policymakers need to be aware of which strains are present in different regions. However, clinical studies of malaria are logistically difficult and expensive to implement. An alternative approach is to study molecular markers in the blood of malaria patients, which can also provide an earlier indication of where resistant parasites have emerged (World Health Organization, 2020). Now, in eLife, Olivo Miotto and colleagues – including Christopher Jacob (Wellcome Sanger Institute) as first author – report a new genetic surveillance platform called ‘GenRe-Mekong’ which monitors the spread of resistant parasites in the Greater Mekong Subregion (Jacob et al., 2021).

Jacob et al. collaborated with various National Malaria Control Programs and scientific partners to collect 9,623 blood samples from patients diagnosed with *P. falciparum* malaria in eight countries (Vietnam, Laos, Cambodia, Thailand, Myanmar, Bangladesh, India and Democratic Republic of Congo). A cutting-edge sequencing technology was then applied to extract and amplify specific genes from the parasitic genome. Jacob et al. analyzed this genetic data for variants which are known to reduce parasites’ susceptibility to the most widely used treatments, including artemisinin (Figure 1). Based on the proportion of variants present in each gene, each sample was then classified as having a ‘sensitive’, ‘resistant’ or ‘undetermined’ response to an antimalarial drug.

**Figure 1.**
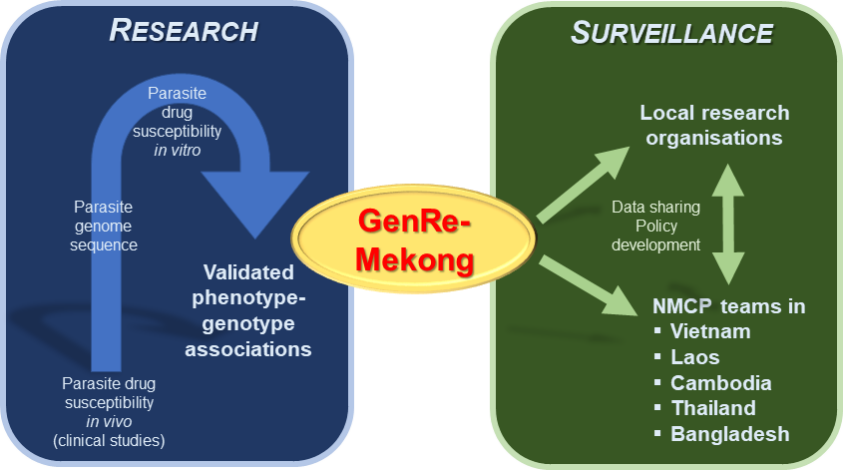
Bridging the gap between research and malaria surveillance in the Greater Mekong Subregion. Previous research studying the genome of parasites has led to the identification of genetic variants which reduce parasites’ susceptibility to antimalarial drugs (blue box, left). Jacob et al. used this data to create a genetic surveillance platform called GenRe-Mekong, which analyzes the blood samples of malaria patients for these genetic variants (green box, right). Data from this platform is then regularly shared with local research organizations and various National Malaria Control Programs (NMCPs) in the Greater Mekong Subregion, who can use this information to help inform their policy decisions for tackling drug-resistant infections.

Data from the genetic surveillance platform were delivered to National Malaria Control Programs as maps which simply describe how the presence and absence of resistant parasites is changing over time. Notably, the team (who are based in the United States, United Kingdom, Vietnam, Laos, Cambodia, Thailand, Bangladesh, Switzerland, Democratic Republic of Congo, France, Myanmar and India) provided concrete examples of how this information can be translated into policy decisions. For example, the database found that parasites less susceptible to artemisinin and one of its partner drugs (originally identified in Cambodia and Thailand) had spread to southern provinces in Vietnam and Laos which were previously unaffected by these resistant strains. This led National Malaria Control Programs in these regions to reassess which frontline therapies to use and where to allocate resources to help combat the rise in drug resistance.

Beyond providing actionable information on the spread of resistance, the approach could also shed light on the complexity of infection, revealing whether a patient was carrying different species or strains of parasites at the same time. It could also be used to infer where the strains detected originated, which could help to reconstruct how parasites resistant to multiple drugs spread to different regions.

Although this platform could rapidly become an essential, complementary strategy for eliminating malaria, additional work is needed to overcome some drawbacks. First, the changes in drug susceptibility caused by the genetic variants is not directly tested, but assumed on the basis of earlier research (Figure 1). Second, the platform will be unable to capture new resistant strains, unless newly discovered variants are continuously added to the GenRe-Mekong database. Last, the current approach is unlikely to be applicable to countries in the African continent. This is because patients are often infected by multiple strains of *P. falciparum* at the same time, which makes it difficult to apply the method across many variant genes and classify the resistance profile. Nevertheless, the work by Jacob et al. demonstrates how genetic data can be a tremendously practical tool that can help policy makers rapidly adapt their treatment strategies in response to rising levels of drug resistance.
